# The Association Between Environmental Factors and Scrub Typhus: A Review

**DOI:** 10.3390/tropicalmed10060151

**Published:** 2025-05-27

**Authors:** Shu Yang, Shu Yang, Yuxiang Xie, Wenjing Duan, Yiting Cui, Ai Peng, Yisheng Zhou, Yibing Fan, Hui Li, Peng Huang

**Affiliations:** 1 Center for Evidence-Based Medicine, Jiangxi Provincial Key Laboratory of Disease Prevention and Public Health, School of Public Health, Jiangxi Medical College, Nanchang University, Nanchang 330019, China; younsoo@163.com (S.Y.); xieyuxiang12123@163.com (Y.X.); jingwend1258@163.com (W.D.); cuiyiting222@163.com (Y.C.); 18589885425@163.com (A.P.); 2 The Collaboration Unit for Field Epidemiology of State Key Laboratory of Infectious Disease Prevention and Control, Nanchang Center for Disease Control and Prevention, Nanchang 330038, China; yangshu@email.ncu.edu.cn (S.Y.); 18079167868@163.com (Y.Z.); fan_yibing@163.com (Y.F.)

**Keywords:** scrub typhus, environmental factors, meteorological determinants, risk factors, review

## Abstract

Scrub typhus is an acute febrile vector-borne infectious disease caused by *Orientia tsutsugamushi* (*O. tsutsugamushi*) and transmitted through the bite of infected chigger mite larvae. Transmission involves complex ecological interactions among vectors, hosts, and environmental factors. Accumulating evidence indicates complex interactions between the scrub typhus incidence and multilevel environmental determinants, encompassing meteorological factors (e.g., temperature, humidity, precipitation, wind speed, sunshine duration, and atmospheric pressure), geographical conditions (e.g., topography, elevation, and landcover), and socioeconomic factors (e.g., economic level, cultural practices, residential conditions, and human behaviors). However, significant discrepancies persist among studies regarding the effect sizes and temporal associations, and the precise mechanisms remain incompletely elucidated. This review synthesizes the evidence on environment–disease relationships, clarifies the methodological inconsistencies, analyzes the potential sources of heterogeneity, and highlights the critical knowledge gaps to inform targeted prevention and control strategies and guide future research priorities.

## 1. Introduction

Scrub typhus is a naturally occurring acute febrile epidemic disease caused by infection with *Orientia tsutsugamushi* (*O. tsutsugamushi*) [1]. The infection is transmitted by the larval stage of trombiculid mites (“chiggers”) [2], primarily from the genus *Leptotrombidium*, with rodents serving as the principal hosts. Patients infected with scrub typhus exhibit complex clinical symptoms, which can be severe enough to cause death [3,4]. In recent years, existing studies have found that the occurrence of scrub typhus may be closely related to a variety of environmental factors, but the results of these studies are not consistent with each other. In this paper, we review the association between environmental factors and the incidence of scrub typhus and sort out the existing research results and controversies, incorporating disease ecology to elucidate how environmental factors affect disease transmission, thereby providing clues or bases for the prevention and control of the disease and further research.

## 2. The Characteristics of Scrub Typhus

### 2.1. Disease Ecology of Scrub Typhus

The transmission ecology of scrub typhus involves complex interactions among the pathogen, vector organisms, hosts, and environmental factors. Scrub typhus is a classic vector-borne zoonotic disease, transmitted to humans through the bite of infected larval-stage chigger mites. The life cycle of chigger mites includes four stages: egg, larva, nymph, and adult [5]. Chigger nymphs and adults do not bite humans. Only the larval stage is parasitic and capable of transmitting the pathogen through skin penetration and feeding [6].

Chigger mites primarily parasitize small mammals, especially rodents. They exhibit relatively low host specificity and often parasitize a wide range of vertebrate species opportunistically [5]. The composition of the dominant vector species varies geographically [2,7]. In the Asia–Pacific region, scrub typhus is mainly caused by *O. tsutsugamushi*, and the principal vectors are *Leptotrombidium deliense* (*L. deliense*), *L. scutellare*, *L. pallidum* and *L. imphalum*, among others. The main host animal in this region is the commensal rodent *Rattus tanezumi* [8]. Within this region, in the northeastern region of India, particularly in Darjeeling, *Schoengastiella ligula* has been identified as an important vector [9]. In South America, particularly in southern Chile, including Chiloé Island and the Aysén region, scrub typhus is primarily transmitted by chiggers of the genus *Herpetacarus*, with small rodents such as *Abrothrix olivacea* and *Abrothrix sanborni* serving as the common hosts [10,11].

The stable circulation of scrub typhus within natural foci relies on the complex ecological interactions among chigger mites, reservoir hosts, and the pathogen.

### 2.2. Clinical Manifestations and Hazards

Patients infected with scrub typhus may present with a variety of clinical symptoms [12], affecting nearly every organ system. Fever, headache, cough, shortness of breath, nausea, and abdominal pain occur in decreasing order of frequency [13]. The most characteristic clinical presentation of scrub typhus is the development of an eschar [14], which typically appears at the site of the chigger bite. An eschar is also a pathognomonic finding for the clinical diagnosis of scrub typhus. The most common complication is acute kidney injury, followed by respiratory issues, cardiac problems, and neurological manifestations. Untreated or inadequately managed scrub typhus can lead to severe systemic complications with multi-organ involvement and potentially fatal outcomes [15]. Studies show that the case fatality rate can be as high as 70% in untreated patients, while it decreases to 1.4% in those receiving appropriate therapy [4]. Recent studies have increasingly emphasized that the variability in the clinical severity of scrub typhus may be partly attributable to differences among *O. tsutsugamushi* strains. Globally, more than 70 strains have been identified, exhibiting considerable genetic and geographic diversity [16]. Clinical observations and experimental studies have suggested that prototype strains such as Karp, Gilliam, and Kato display distinct virulence profiles. Further evidence from animal model experiments has demonstrated that Karp strains possess greater virulence than strains such as Gilliam, leading to more severe lung pathology [17]. In addition, Karp-like strains have been associated with higher mortality rates and a greater incidence of severe complications [18]. These strain-related differences in virulence may contribute to the heterogeneous clinical manifestations observed in scrub typhus [19].

### 2.3. Global Epidemiology

Scrub typhus demonstrates distinct spatiotemporal heterogeneity and demographic variation in its global transmission patterns. Regarding the temporal distribution, the high incidence period is distinctly geographic globally. For example, in India, the peak incidence is concentrated in the monsoon and post-monsoon seasons from July to February [19]; in northern Vietnam, the high incidence is mainly in the summer months, whereas there is no clear seasonality in the southern part of the country [19]; in Japan, the peak incidence occurs in November and May [20]; and in South Korea, the incidence starts to increase from September and reaches its peak in October [21]. In China, scrub typhus is predominantly of the summer and fall types [22], with the high incidence of the summer type occurring from May to November and the high incidence of the fall type occurring from October to November. Some areas, such as central Jiangxi and parts of Hunan, show a staggered distribution of summer-type and fall-type infected regions [23]. This seasonality largely corresponds to the activity period of chigger larvae, which are typically active from early spring through late fall [2].

In terms of the spatial distribution, scrub typhus demonstrates pronounced geospatial clustering. The “Tsutsugamushi triangle” in the Asia–Pacific region, which extends from Japan in the east to Afghanistan in the west, from northern Australia in the southeast to the north, and from the Far East coast of Russia in the northeast, is the most prevalent area of scrub typhus worldwide [24]. While the historical transmission has concentrated within this zone, emerging foci have increasingly been identified beyond its classical boundaries. Notably, a study [11] conducted in southern Chile confirmed the existence of a novel human pathogen, *Candidatus Orientia chiloensis*, the third *Orientia* species recognized as capable of infecting humans, after O. tsutsugamushi and *Candidatus* Orientia chuto. This pathogen is mainly transmitted by local chiggers of the genus *Herpetacarus* (e.g., *Herpetacarus eloisae*), rather than the genus *Leptotrombidium*, which is common in Asian scrub typhus. Scrub typhus is no longer confined to the Asia–Pacific region, with region-specific vectors and hosts potentially sustaining independent transmission cycles in newly affected geographic regions.

Regarding the population distribution, demographic analyses reveal occupation-specific and sex-discrepant scrub typhus risks. Both domestic and international studies have consistently demonstrated that farmers are at high risk of scrub typhus, mainly because their occupational activities increase the likelihood of exposure to bites from infected chigger larvae, the primary transmission route of scrub typhus. However, individual studies have also reported that homemakers, students, and children experience a higher incidence of the disease in certain regions [25]. Regarding gender, existing studies from domestic and international settings report conflicting findings; some studies suggest that females are at higher risk of infection [26], while others have shown that cases of scrub typhus are more common in males [27].

In summary, the epidemiological characteristics of scrub typhus align closely with the ecological requirements of its chigger mite vector. The transmission peaks occur during the warm and humid summer and autumn seasons. Additionally, high-risk populations are primarily found among agricultural workers and in geographical areas where significant interaction between humans and vegetation is present.

## 3. Association of Natural Environmental Factors with Scrub Typhus

Scrub typhus is a vector-borne zoonotic disease, with the vector role fulfilled by larval chigger mites. Female chiggers transmit *O. tsutsugamushi* to the next generation of chigger larvae through transovarial transmission, and chigger larvae form the natural chigger infection cycle by biting rodents. The occurrence of scrub typhus in humans is mainly dependent on the exposure of *O. tsutsugamushi*-infected vector chiggers to the population. The key factors in the prevalence of scrub typhus are the overlap between the breeding sites of the vector chiggers and the habitats of the population and the overlap between the environments in which the vector chiggers live and human activities, as well as the ecological interactions between the vector, hosts and environmental conditions. A nationwide study [28] in Thailand identified a significant positive correlation between the chigger species richness and the human scrub typhus incidence. Existing studies [2,28] suggest that various natural environmental factors significantly influence the survival, development, and reproductive success of chigger larvae (vectors) and rodent reservoirs (primary hosts). Chigger mites, particularly species within the genus *Leptotrombidium*, act as the primary vector for scrub typhus transmission. These vectors exhibit strong sensitivity to environmental conditions, especially temperature and humidity [29,30]. The vegetation type and land cover also influence chigger habitats [28]. Natural environmental factors, mediated through vector and host ecology, play a pivotal role in shaping the spatiotemporal transmission dynamics of scrub typhus. The varying sensitivities of different chigger mite species and host animals to environmental conditions further contribute to the pronounced spatial and temporal heterogeneity observed in the disease transmission.

### 3.1. Meteorological Factors

Climate change profoundly influences the incidence of infectious diseases, and scrub typhus is no exception. As a climate-sensitive vector-borne disease, scrub typhus exhibits incidence patterns modulated by meteorological parameters, including the temperature, humidity, and precipitation. These factors directly affect the living environment of vectors and hosts or indirectly change the distribution of scrub typhus by affecting the distribution of vegetation [27]. On the one hand, climatic factors significantly influence the development of chigger larvae; on the other hand, extreme weather events such as heatwaves and heavy rainfall alter human behavioral patterns, thereby modifying the human–vector contact opportunities.

#### 3.1.1. Temperature

Temperature is recognized as a primary climatic driver influencing scrub typhus transmission. One researcher [31] employed a generalized additive mixed modeling framework to examine the association between the spatiotemporal patterns of scrub typhus and climatic determinants. Separately, investigators [32] analyzed the epidemiological dynamics of scrub typhus in southern China (2007–2017) using a boosted regression tree (BRT) integrating spatial autocorrelation structures. Both studies conclusively identified the temperature variability and relative humidity as the predominant drivers governing the disease transmission mechanisms. Another Chinese study [33] employed a distributed lag nonlinear model (DLNM) combined with a Poisson regression to analyze the nonlinear exposure–response relationships and lagged effects of meteorological factors on the scrub typhus incidence, which showed that the association between the temperature and the incidence of scrub typhus could be described as an initial-elevated-descendent pattern. As the temperature increased, the incidence of scrub typhus first remained stable. Gradually, it increased to its peak and proceeded further into a decrease. The incidence of scrub typhus peaked when the mean temperature reached 24.5 °C, with a cumulative relative risk of 10.10 (95% CI: 7.45–13.60). In a study conducted in Guangzhou, China [34], using a multivariate negative binomial regression model, each 1 °C increase in temperature corresponded to a rise of 14.98% (95% CI: 13.65–16.33%) in the monthly number of scrub typhus cases. In contrast, an Indian study [35], using geographically weighted regression and Spearman’s correlation analysis, reported an 18.8% (95% CI: 13.2–24.1%) reduction in the monthly incidence per 1 °C increase in the mean temperature, contradicting the prevailing epidemiological patterns. The Indian researchers hypothesized that the summer temperatures in India (averaging 31 °C) exceed the optimal thermal range for chigger mite reproduction and survival (20–30 °C). Consequently, extreme heat may suppress vector populations, while a slight decrease within the optimal range may enhance chigger mite fitness, resulting in an inverse relation between the average temperature and the disease incidence. These contrasting findings suggest that the baseline climatic conditions critically shape the relationship between the temperature and the scrub typhus incidence across different regions and may further contribute to the observed heterogeneity between studies.

Furthermore, regarding the reported linear and nonlinear associations between the temperature and the scrub typhus incidence across the included studies, a certain degree of heterogeneity is evident. The primary source of this heterogeneity likely stems from the methodological differences among studies. For instance, some studies (such as those conducted in Guangzhou [34] and India [35]) employed conventional regression models, generally assuming a linear relationship between the variables and the disease incidence, whereas DLNMs can simultaneously model both nonlinear and lagged effects. These methodological discrepancies fundamentally contribute to the variability in the effect size estimates across studies.

In addition, the heterogeneity in study design is another major factor, including differences in the definition of the outcome indicators, strategies for controlling confounding factors, and the temporal resolution of analysis. All these differences may influence the effect size estimation. Nevertheless, despite the observed heterogeneity, the overall trend across existing studies consistently supports the conclusion that temperature changes significantly influence the scrub typhus incidence, with most findings indicating a positive association.

Furthermore, emerging research has identified the critical role of the land surface temperature (LST) in the scrub typhus transmission dynamics. A study in Guangzhou, China [36], employing a distributed lag nonlinear model, demonstrated a 3.8% increase in the weekly scrub typhus risk per 1 °C LST elevation. Similarly, a retrospective spatiotemporal analysis in Hainan, China [37], identified an incremental effect of 3.279 cases per 1 °C temperature rise (*p* = 0.005). However, conflicting evidence has emerged from South Korea, where a Bayesian spatiotemporal model [38] showed an inverse association—an autumnal LST elevation by 1 °C reduced the relative risk to 0.938 (95% CI: 0.903–0.977). In response to this paradoxical phenomenon, the possible mechanism involves significant spatial and temporal heterogeneity and geographic differences between the surface temperature and the incidence of scrub typhus. It may also be related to differences in the spatial scales of the data collection, and follow-up studies need to be conducted more on the surface temperature, which is a controversial factor.

Dual mechanisms govern the temperature-mediated scrub typhus incidence: chigger mite ecology and human exposure patterns. As poikilothermic organisms, chigger mites exhibit temperature-dependent life-history traits, with the thermal conditions critically regulating their developmental cycles, oviposition rates, longevity, and questing behavior [1]. Within optimal thermal ranges, the chigger egg hatching and larval development rates positively correlate with the temperature elevation. However, exceeding the thermal thresholds significantly suppresses the hatching efficiency. Mite populations exhibit significant interspecific variations in their thermal adaptation strategies. *L. deliense*, the predominant vector in southern and southeastern China, demonstrates maximal field hatching rates at 23 ± 1 °C [39]. *L. scutellare* displays distinct seasonal adaptation patterns, demonstrating enhanced cold tolerance and drought resistance during the winter–spring activity periods [40].

From the behavioral ecology perspective, most chigger species exhibit maximal mobility at 22–24 °C [41], while their host animals demonstrate preferred activity temperatures 3–5 °C higher (27–29 °C) [42]. In terms of the human exposure risk, ambient temperature also shows a nonlinear relationship with human behavioral patterns; as temperatures gradually increase, residents are outdoors more frequently, usually accompanied by less clothing being worn, more skin exposure, and thus more exposure to chiggers; however, excessively high temperatures may decrease outdoor activities, consequently reducing the scrub typhus infection risks.

#### 3.1.2. Humidity

Environmental humidity is a critical determinant of chigger mite prevalence and geographic distribution [34], as well as a key factor in rodent reservoir reproduction [43]. Chigger mites rely primarily on atmospheric moisture for survival. A study in Vellore district, India, analyzed 15 years of chiggers data as well as climatic data [35] and found that each 1% increase in the mean relative humidity was associated with a 7.6% (95% CI: 5.4–9.9%) rise in monthly scrub typhus cases. A Chilean study noted that chiggers thrive in environments with relative humidity > 50%, but their activity significantly declines when the humidity exceeds 75–80% or falls below 50% [43]. Reduced humidity suppresses or halts oviposition rates in adult chiggers, while elevated humidity enhances larval hatching success. This suggests that moderate humidity increases facilitate the moist microhabitats essential for chigger reproduction and development. A study in Guangzhou, China [36], utilizing the DLMN, identified a lagged association between the relative humidity variations and the scrub typhus incidence: a 10.00% increase in relative humidity elevated the disease risk by 8.5% after a 4-month lag. This lagged effect may be attributed to the temporal disjunction between the meteorological influences, the chigger life cycle (approximately 2–3 months), and the disease incubation period (averaging 10–12 days). However, the observed 4-month lag period needs to be interpreted with caution, as it exceeds the typical life cycle duration of scrub typhus. As chiggers parasitize only one host per life stage and transmit *O. tsutsugamushi* to subsequent generations via transovarial transmission by female mites [44], the larval infection rates and population density are predominantly determined by preceding generations’ infection prevalence and the population density of the previous generation [45]. This raises the possibility that longer-term climatic conditions may indirectly influence the scrub typhus risk by affecting the vector and host dynamics across multiple generations rather than through direct effects on a single cohort. Furthermore, the humidity and atmospheric pressure fluctuations during the preceding 1–2 months critically govern the egg-hatching success and early larval survival, creating cascading effects on subsequent transmission cycles.

#### 3.1.3. Precipitation

Precipitation is a key meteorological factor affecting rodent populations’ size. The scrub typhus incidence peaks during rainy seasons, as moderate precipitation promotes vegetation growth, creating optimal habitats for chigger mites. It facilitates rodent survival directly or indirectly, while lush vegetation provides abundant food resources for mite reproduction and survival. A study in Thailand [46] observed accelerated dispersal behavior of *L. deliense* during rainfall, resulting in higher mite attachment rates to rodents in rainy seasons compared to other periods. However, a study in China [33] observed a decrease in the incidence of chiggers when the precipitation exceeded a certain threshold. A generalized additive mixed model [31] revealed a nonlinear inverted U-shaped relationship between precipitation and the case counts, with a negative correlation threshold at 100 mm/month—the incidence decreased when the monthly precipitation surpassed this value. This pattern aligns with findings from a nationwide time-series analysis in South Korea [47]. From the vector ecological and human behavioral perspectives, only larval-stage chiggers parasitize hosts, while other life stages inhabit soil and vegetation [2]. During dry seasons, chiggers are mostly found underneath brush and shady areas, whereas in the wet season, chiggers are usually found in tall grass and dense vegetation. Human activities such as standing still or lying in tall grass give them more time to climb onto a person and bite [28]. Excessive precipitation destroys the microhabitat where chiggers breed, resulting in a significant reduction in mite populations, and secondly, heavy rainfall limits human outdoor activities, decreasing the exposure risks. In addition, a study [33] linked scrub typhus outbreaks to short-term extreme precipitation events. Torrential rains and floods force rodent displacement from burrows into human settlements, amplifying human–rodent contact [48]. Therefore, post-flood periods frequently exhibit marked increases in the scrub typhus incidence.

#### 3.1.4. Wind Speed

There are limited studies on the relationship between wind speed and scrub typhus. An ecological study conducted in Fujian Province, China [48], demonstrated that strong wind conditions reduce the scrub typhus infection risks, though the underlying mechanisms remain unclear. It was speculated that high wind speeds may hinder the survival and development of chigger larvae by physically displacing them from suitable microhabitats. Eggs or newly hatched larvae may be blown away from favorable moist vegetation or soil surfaces, reducing the chances of successful attachment to hosts and subsequent transmission. In addition, strong winds may discourage outdoor human activities, further decreasing the opportunities for human–chigger contact [2]. However, most studies [36,48,49,50,51] consistently report positive correlations between the wind speed and the scrub typhus incidence. A focused investigation in southern Jiangxi Province, China [52], revealed that the average wind speed exhibited stronger positive associations with the disease incidence in southern regions compared to northern areas. Notably, significant interactive effects were identified between the average wind speed and the forest coverage (*q* = 0.356). Moderately high wind speeds are accompanied by large areas of forested land cover, and driven by wind speeds, chiggers on forest foliage are able to spread over long distances, which in turn leads to an increased risk of infection. Controversy still exists between studies, and future research could focus on the impact of the wind speed on the ecological characteristics and epidemiological effects of chigger mites, systematically elucidating the underlying mechanisms associated with the scrub typhus incidence.

#### 3.1.5. Sunshine

Existing studies [47,53,54] indicate that a prolonged sunlight duration constitutes a risk factor for scrub typhus. A positive correlation exists between the case numbers and the sunlight duration, with each additional hour of sunlight corresponding to a 0.17% or 0.54% increase in monthly scrub typhus cases. This association may be attributed to extended daylight hours promoting a longer period of human outdoor activities, thereby amplifying contact with chigger-infested vegetation and elevating exposure risks. A previous study [34] found that chiggers’ activity is associated with microclimate-driven circadian rhythms, with the activity being most active during the afternoon to sunset hours, dropping to low levels in the evening and remaining so until sunrise. The timing of chiggers’ activity highly overlaps with that of human activity, potentially increasing the chances of chiggers infecting humans. Together, these behavioral synchronizations between vectors and humans during extended daylight periods may synergistically amplify the scrub typhus transmission risk.

#### 3.1.6. Atmospheric Pressure

Research on the association between atmospheric pressure and the scrub typhus incidence remains insufficient. An early investigation in Guangzhou, China [34], first examined the atmospheric pressure effects on the scrub typhus incidence in southern China, revealing negative correlations between the monthly atmospheric pressure elevation and disease occurrence. The mechanism may be because high atmospheric pressure environments are usually accompanied by lower humidity and temperature with fewer hours of sunshine, and these meteorological conditions are not favorable for mite survival, which may reduce the number of scrub typhus cases. However, subsequent negative binomial regression analyses [48] demonstrated that each 1 kPa increase in atmospheric pressure over the preceding three months corresponded to 30% monthly case escalation. Similar patterns were validated in hyperendemic regions of Guangxi and Yunnan provinces [55]. These contradictory findings suggest potential lagged effects, where short-term (concurrent month) and prolonged (2–4 months lagged) atmospheric pressure influences exhibit diametrically opposed epidemiological impacts. Long-term cumulative changes in the atmospheric pressure may affect the scrub typhus transmission risk by regulating the population dynamics of hosts or vectors. However, studies on the mechanisms by which changes in atmospheric pressure affect ecological processes in hosts or vectors are still limited and need to be further systematically explored. This highlights the necessity of incorporating meteorological factors’ dynamic cumulative effects and temporal lags when investigating atmospheric pressure–scrub typhus relationships.

### 3.2. Geographical Factors

#### 3.2.1. Topography and Geomorphology

Topographic heterogeneity modulates the scrub typhus distribution through vegetation-mediated ecological pathways. A study in Fujian Province, China [56], identified Nanping City, a hyperendemic area located in the upper reaches of the Min River, as a region where dense forests, shrublands, grasslands, and riparian systems create optimal habitats for chigger mites and reservoir hosts, driving high disease transmission. Indian research [57] documented a higher scrub typhus incidence in hilly regions below 1000 m elevation compared to plains, attributed to the ecological suitability of mountainous areas characterized by complex vegetation and hydrology for vector–host survival. In contrast, studies in China’s Hainan and Anhui provinces [37,58] reported case clustering in flat plains. The topographic simplicity of these areas facilitates dense human settlements and frequent social interactions, which may enhance the transmission efficiency through increased human–environment contact despite suboptimal chigger habitats. This paradox highlights the dual influence of topography: ecologically complex terrains favor vector proliferation, while human-dominated plains amplify exposure risks through demographic factors.

#### 3.2.2. Elevation

Recent research [59] has shown that altitude has been clearly identified as a key geographic determinant of the scrub typhus distribution in southern China and that it can indirectly influence the disease incidence by modulating the microclimate and vegetation cover. A study in Jiangxi Province, China [52], found that as the vegetation became denser with increasing altitude, the incidence of scrub typhus increased. As the altitude increases beyond a specific limit, lower atmospheric pressure, lower temperatures and humidity, and reduced vegetation density are detrimental to chigger growth and activity. High-altitude regions typically exhibit lower population density and limited agricultural cultivation, constraining the disease transmission pathways and resulting in reduced scrub typhus incidence. The biodiversity of scrub typhus communities and the distribution of small mammals fluctuate significantly with the altitude. For example, in Yunnan Province, China, where the incidence of scrub typhus is high [60], the species richness of hosts and chiggers showed a gradual increase and then a decrease in species richness with the change of altitude gradient. The mid-altitude region, which combines suitable microclimatic conditions and mixed vegetation types, is the second highest risk area for disease transmission [37].

Recent ecological studies [30,61] have revealed notable differences in the environmental preferences and host associations of chigger mite species implicated in the transmission of scrub typhus in China. *L. rupestre* tends to inhabit high-latitude and high-altitude regions, particularly in western Sichuan, where it is predominantly found parasitizing wild rodents such as *Alexandromys oeconomus* [30]. This species shows a strong host preference and high infestation rates under alpine conditions. In contrast, *L. deliense* and *L. imphalum* are typically associated with low-latitude, low-altitude environments. Both species are frequently found parasitizing the commensal rodent *Rattus tanezumi*, which commonly inhabits human settlements and agricultural landscapes [7]. *L. imphalum* shows the highest abundance in subtropical zones (21–22° N) and exhibits a marked decline in density above 2500 m [7]. These ecological distinctions suggest that the distribution and transmission potential of scrub typhus vectors are shaped by the elevation, latitude, land use, and host availability, contributing to regional variations in the disease risk and necessitating tailored surveillance strategies. Although these species belong to the same genus, their spatial distributions are clearly ecologically complementary—each occupies a different altitude and habitat type, and thus may pose a risk of transmission of scrub typhus at different altitudes. This difference in ecological adaptation further contributes to the regional variation in the incidence of scrub typhus across the altitudinal gradient due to differences in the dominant vector species in each region.

#### 3.2.3. Landcover

The incidence of scrub typhus demonstrates a close association with the landcover. Disease transmission depends on the geographical distribution of chigger mites [41,62], with distinct vegetation types providing varied habitats and breeding grounds that indirectly influence the mite population dynamics and disease transmission risks. The Normalized Difference Vegetation Index (NDVI), a key indicator of vegetation health and coverage, shows that regions with higher NDVI values typically sustain denser vegetation. These areas offer abundant food resources and concealed habitats for both mites and their hosts, consequently exhibiting elevated scrub typhus incidence [38,47]. Furthermore, research indicates a three-month lagged effect of increased vegetation coverage on the infection risk [55]. Notably, in southern India [63], maintaining NDVI values within the 0.6–0.8 range for four consecutive weeks was identified as a critical vegetation-related predictor of scrub typhus outbreaks. Moreover, the NDVI demonstrates significant interaction effects with the mean temperature and precipitation, where synergistic effects between high vegetation coverage and warm, humid climates substantially enhance mite reproduction and disease transmission [47,52].

Moreover, the NDVI provides a general reflection of the vegetation status, whereas specific vegetation cover types offer more detailed insights into ecological habitats supporting vector proliferation. However, caution is warranted when interpreting the NDVI–disease associations across different regions and seasons, as the vegetation cover types and NDVI values can vary markedly over time and administrative boundaries, potentially introducing spatial and temporal heterogeneity into the observed relationships. In addition to the NDVI, distinct vegetation cover types differentially impact the scrub typhus incidence. Early research [64] identified disease clustering in ecotone habitats characterized by transitional vegetation, including forest edges, riparian shrublands, and post-deforestation areas. Subsequent studies [65] revealed higher scrub typhus prevalence in secondary vegetation zones dominated by dense vegetation such as grasslands and shrubs compared to primary vegetation. A Chinese study integrating vegetation categories into boosted regression tree models [66] determined shrub coverage to be the most critical vegetation determinant, contributing 20.84% to the spatial distribution predictions, followed by farmland (12.10%), grassland (6.74%), and forest (6.41%). Shrub exceeding 2% coverage triggers exponential risk escalation as core chigger mite habitats. Another investigation [48] quantified a 75% case increase per 0.1% shrub coverage increment. Farmland emerges as a secondary risk factor, with areas exceeding 20% coverage exhibiting a significantly elevated risk due to the intensified agricultural activities and exposure during the summer–autumn farming seasons. However, seasonal variation in vegetation phenology and differences across administrative boundaries may lead to variability in the associations between farmland and the disease incidence. Water bodies and wasteland demonstrate contrasting epidemiological effects: 1% water coverage expansion reduces the monthly cases by 4%, whereas 1% wasteland increase elevates the cases by 37% [48]. Further ecological studies corroborate these findings. A field study [8] reported that *Rattus tanezumi* carried a greater diversity and higher abundance of chigger mite species in outdoor habitats such as farmland, grasslands, and shrublands, which may be related to differences in the vegetation structure, humidity, and small mammal community composition. Chigger mites can inhabit a wide range of environments, from grasslands to swampy areas, but are most commonly found in grassy or scrubby vegetation, shaded locations, leaf litter, rotten logs, and tree stumps. The pathogen itself has been detected across diverse habitats, ranging from semi-urban parks and gardens to plantations, sandy beaches, forests, and alpine meadows at elevations up to 3200 m [2]. Moreover, *L. deliense* was identified as the dominant species in the flatland and indoors, while *Gahrliepia longipedalis* prevailed in the mountainous and outdoor areas [7]. These findings underscore the ecological role of vegetation in shaping the distribution of chiggers and hosts and the patterns of human exposure risk. Since vegetation is relatively controllable, this research can help identify areas at high risk of infection, allowing more precise control measures to be taken.

## 4. Socio-Environmental Factors

### 4.1. Socioeconomic Environment

#### 4.1.1. Economic Development Level

The relationship between the gross domestic product (GDP), a key comprehensive indicator of regional and national economic development, and the scrub typhus incidence remains contentious. Zheng et al. [32] employed boosted regression tree models in southern China’s high-risk areas, revealing nonsignificant GDP contributions to the predictive models. Conversely, a Qingdao-based study [67] using an identical methodology identified the GDP as the foremost predictor, potentially attributable to the minimized meteorological and vegetation variation at more minor spatial scales where socioeconomic factors dominate epidemiological drivers. Most studies concur that the GDP lacks substantial independent explanatory power for transmission dynamics, exhibiting complex covariation patterns with other determinants [32]. Spatial regression analyses in southern Jiangxi Province [52] demonstrated economic factors’ indirect operation through the interaction effects (*q* = 0.357) between the GDP and the grassland coverage, constituting the primary explanatory variable for spatial incidence heterogeneity. Higher GDP regions typically feature enhanced public health infrastructure, including improved laboratory diagnostic capabilities, which facilitate better detection and reporting of scrub typhus cases. Conversely, in economically disadvantaged regions, there is often a lack of capacity to perform confirmatory laboratory testing. Due to resource constraints, empirical antibiotic treatment is frequently administered without a definitive diagnosis, resulting in substantial underdiagnosis and underreporting of scrub typhus cases. These factors may collectively lead to an underestimation of the true burden of the disease. In addition to the detection biases, higher GDP regions may also experience elevated risks through population density intensification, expanded transportation networks, and tourism activities that amplify the human–mite contact frequency. Thus, both detection biases and real exposure differences may confound the observed associations between the GDP levels and the scrub typhus incidence. Moreover, the broader environmental consequences of economic growth may indirectly influence the scrub typhus transmission dynamics. Economic growth inevitably leads to environmental pollution, with excessive greenhouse gas emissions exacerbating global warming. Warmer climates create favorable conditions for rodent and mite proliferation, thereby intensifying the infectious disease risks. In addition, eco-friendly policies advocated by the government, such as reducing industrial land use and restoring forests and wetlands, have also contributed to the formation of new sources of infection.

#### 4.1.2. Urbanization

Along with increasing urbanization, the geographic distribution of scrub typhus is gradually expanding and has progressively spread from traditionally endemic areas such as agricultural and mountainous regions to urban areas [68]. In Hainan Province, China, rapid urbanization has discharged nitrogen- and phosphorus-rich nutrients into ecosystems, potentially promoting vector proliferation [37]. Such environmental changes may indirectly facilitate the spatial diffusion of the transmission risk. Urbanization contributes to the spatial diffusion of transmission risks. Between 2010 and 2013, more urban-indigenous cases were reported in Seoul, South Korea [68]. However, urbanization does not universally increase the risk of scrub typhus among urban residents. A study conducted in Thailand [28] found that the chigger species richness decreases gradually with an increasing intensity of human land use. As a result, vectors and hosts are forced to seek new habitats, often relocating to peri-urban green spaces, thereby giving rise to new patterns of infection risk. In addition, a study [69] showed that peri-urban areas are one of the most critical risk factors influencing the occurrence and spread of scrub typhus in Nepal. In a study from India [57], scrub typhus mainly concentrates in the city’s central location, but scattered cases of scrub typhus have also accumulated into more than 100 cases in suburban locations on the urban fringes. In recent years, the establishment of urban natural parks and preservation of peri-urban green spaces, intended to enhance environmental sustainability, inadvertently created favorable habitats for chigger mites and rodent reservoirs [48]. Guangzhou City research revealed that 20–40% of annual urban cases occur among individuals classified as farmers residing in newly urbanized peri-urban zones, where residents continue agricultural activities [48]. Similarly, a study [70] in Vientiane, Laos, found that the scrub typhus seropositivity was significantly higher among adults living in the periphery (28.4%) than in the central zone (13.1%) of Vientiane. As urbanization progresses, chiggers and their hosts increasingly infiltrate urban parks and suburban zones, shifting transmission risks. Thus, the interaction of urban population growth with the environmental and socioeconomic conditions influences the dynamics of vector-borne diseases and may increase the risk of scrub typhus infection among urban populations through enhanced chigger–human contact.

### 4.2. Sociocultural Environment

Multiple studies have revealed a complex relationship between education and literacy levels, the perception of scrub typhus, and the risk of infection. A case-control study in Beijing, China, identified a significantly higher infection risk among individuals with ≤6 years of education, suggesting low education to be a critical risk factor [71]. This aligns with Devamani et al.’s survey of 1353 Indian residents, demonstrating significant education–disease associations [51]. A study in Thailand [72] also found that the risk of infection in uneducated study participants was 3.46 times (95% CI = 1.93−6.21) higher than in those with basic education. A study based on Bayesian spatiotemporal hierarchical modeling found [59] that illiteracy is a major social factor in terms of the scrub typhus risk in northern China (*q* = 0.844), which is associated with a high proportion of the elderly population, low urbanization, and weak level of healthcare resources, which further exacerbate the incidence of scrub typhus. Further studies have revealed that the education level may affect individuals’ commitment to disease prevention by influencing health knowledge acquisition. A case-control study in South Korea showed [73] that the uninfected group had a more comprehensive knowledge of scrub typhus epidemiologic seasons, clinical manifestations, and other aspects of the disease, and that the educated individuals possessed higher cognitive levels. Similarly, research in Yunnan Province, China [74], also found that residents with higher education levels had significantly higher knowledge–attitude–practice scores regarding scrub typhus, indicating that educational improvement may promote preventive behaviors. A study [75] conducted in Niigata Prefecture, Japan, focusing on vector-borne disease, found that individuals with higher knowledge levels were more likely to adopt preventive behaviors; however, there was a clear “knowledge–practice gap” identified among the respondents. Approximately 84% of respondents recognized that reducing skin exposure could prevent infection, but only about 70% actually practiced it. Additionally, while 38% were aware of the use of repellents, only 31% reported actual usage. Contrasting evidence exists. Park et al.’s study in South Korea [76] found no statistically significant correlation between the seroprevalence of scrub typhus in South Korea and educational attainment. The results of a case-control study conducted by Wei et al. [77] in Guangzhou City, China, similarly showed that there was no significant correlation between educational attainment and the incidence of scrub typhus. Despite these conflicting findings across studies, cumulative evidence suggests educated populations generally possess enhanced disease knowledge and are more likely to adopt protective behaviors, collectively reducing the infection susceptibility.

### 4.3. Human Behaviors and the Residential Conditions

#### 4.3.1. Human Behaviors

In the context of globalization, the high-frequency movement of people and goods, especially population migration, the development of the tourism industry, and the increase in international trade activities have led to a global expansion of the prevalence of scrub typhus, with the number of cases continuing to grow. Through global trade, the vector chiggers and their hosts can be spread to expand to new areas through transportation, trade in plants and animals, and other means. In addition, global warming due to human activities has exacerbated the reproduction of vector chiggers and their hosts, thus allowing the global spread of infectious diseases that have traditionally been localized [12].

A study [2] indicated that human behavior is likely a more critical risk factor for acquiring scrub typhus than previously recognized. Chiggers are unlikely to bite humans without sufficient opportunities for attachment. Although infected vectors persist year-round, fluctuations in case numbers are likely driven by changes in human exposure patterns.

Individual behavior, especially outdoor activities, work practices, and the adoption of protective measures, directly affects the chances of contracting scrub typhus. Current evidence identifies frequent outdoor activities as a key risk factor for scrub typhus [71,78]. Human encroachment into chigger habitats (forests, grasslands, shrublands) during recreational or occupational activities elevates mite contact risks. In addition, agricultural workers face heightened vulnerability due to prolonged outdoor exposures during harvesting, weeding, and planting activities that unavoidably increase vector encounters. Agricultural activities are also specific to the development of scrub typhus in different regions. A study [79] among aboriginal subgroups in West Malaysia reported varying *O. tsutsugamushi* antibody prevalence, with higher exposure risks observed in individuals engaging in subsistence farming. In Bhutan [26], cultivation of cardamon is a high-risk behavior in relation to scrub typhus infection; in the central region of Vietnam [80] and in South Korea [73], people engaged in grazing and logging in forests and hilly areas are at a higher risk of infection.

In many cases, individuals are often exposed to a high risk of scrub typhus infection for long periods of time due to their occupational or living conditions. However, whether or not to take protective measures is a behavioral choice that is within the control of the individual. In addition to minimizing high-risk outdoor activities, the use of chemical insect repellents (e.g., products containing ingredients such as DEET or Picaridin) has been shown to significantly reduce the risk of contracting scrub typhus [81,82]. The mechanism of protection is primarily reducing the attachment of chigger larvae to the surface of the skin, thereby reducing the chance of bites occurring [83], which are a key component of the transmission of scrub typhus. Similarly, wearing protective clothing is effective in reducing the risk of bites. Consistent evidence from epidemiological studies supports the protective role of personal behaviors. A Vietnamese case-control study [80] confirmed that the consistent use of gloves, long-sleeved clothing, and repellents during outdoor activities markedly reduces exposure risks. Further studies [72] have suggested that gloves may be an independent protective factor in the development of scrub typhus, with those who did not wear gloves at work having a risk of disease infection 2.12 times higher than those who wore matching gloves on a daily basis (95% CI: 1.20–2.84), and that gloves reduce direct skin contact with scrub typhus, thereby reducing the risk of infection.

Epidemiological evidence confirms significant associations between individual behavioral patterns and disease infection risks. Targeted preventive measures against these established risk factors, with focused behavioral corrections in high-risk populations, can effectively reduce the infection risks.

#### 4.3.2. Residential Conditions

Human occupational and residential environments significantly influence the risk of scrub typhus infection. Scrub typhus vectors and reservoirs, including chigger mites and small mammals, prefer habitats with tall grass, shrubs, low canopies, and abundant leaf litter, including areas around homes and filled with poor sanitation and vegetation overgrowth [84]. First, geographical proximity serves as a critical determinant, with multiple case-control studies [71,73,80,85,86] demonstrating heightened disease prevalence among populations residing near chigger mite habitats, particularly vegetation-rich areas, farmland-intensive zones, and regions adjacent to water sources. Second, residential sanitation conditions crucially mediate vector control outcomes, as inadequate environmental hygiene management promotes chigger mite proliferation. Empirical evidence identifies synergistic risk factors: poor household sanitation [80], frequent rodent sightings within dwellings [80,87], elevated ambient humidity [73], absence of indoor sanitation facilities [87], and yard storage of firewood or crops [26,71,88]. These factors collectively increase human–vector interaction opportunities, thereby amplifying the disease transmission risks.

## 5. Conclusions

Environmental factors are associated with the scrub typhus incidence, with meteorological changes, geographical conditions, socioeconomic environments, and individual behaviors potentially influencing the disease occurrence. Current research reveals ongoing controversies regarding specific environmental associations. Environmental factors may affect the ecological dynamics of the disease and human behavior patterns through both direct and indirect pathways, thereby altering the transmission dynamics. Moreover, the heterogeneity across studies—including differences in geographical regions, research designs, methodological approaches, and the ecological characteristics of vectors and hosts—may also contribute to the inconsistency of the findings. Further studies are required to clarify these complex interactions.

## Data Availability

Not applicable.

## Abbreviations

The following abbreviations are used in this manuscript:

DLNM	Distributed lag nonlinear model
GDP	Gross domestic product
*L. deliense*	*Leptotrombidium deliense*
*L. imphalum*	*Leptotrombidium imphalum*
*L. pallidum*	*Leptotrombidium pallidum*
*L. scutellare*	*Leptotrombidium scutellare*
LST	Land surface temperature
NDVI	Normalized Difference Vegetation Index
*O. tsutsugamushi*	*Orientia tsutsugamushi*
